# Establishment of a finite element model of supination-external rotation ankle joint injury and its mechanical analysis

**DOI:** 10.1038/s41598-022-24705-5

**Published:** 2022-11-22

**Authors:** Xin Zhang, Pinliang Xie, Weirong Shao, Ming Xu, Xiaoping Xu, Yong Yin, Lan Fei

**Affiliations:** Department of Orthopedics, Jiading District Central Hospital Affiliated Shanghai University of Medicine & Health Sciences, 1 Chengbei Road, Shanghai, 201800 China

**Keywords:** Diseases, Trauma, Bone, Tendons

## Abstract

By establishing a three-dimensional finite element model of ankle injury arising from supination and external rotation, the stress characteristics of the posterior malleolar surface can be obtained, and analysis of the corresponding stress on the lateral ankle can be conducted. Thin-layer computed tomography images of normal ankle joint in the supination and external rotation nonweight-bearing states was selected, to construct a three-dimensional data model of each ankle joint. A load was applied to examine different ankle joint stress values and pressure distributions on the surface of the posterior ankle joint. A 600 N vertical compressive and 10 Nm internal rotation load was applied in Stage III (removing the anterior tibiofibular ligament and the posterior tibiofibular ligament) of SER (supination-external rotation). When the lateral malleolar was intact, the maximum stress (132.7 MPa) was located at the point of attachment of the posterior tibiofibular ligament to the fibula, and the maximum pressure of the posterior malleolar surface was lower than 4.505 MPa. When a lateral malleolar fracture was present, the maximum stress (82.72 MPa) was located on the fibular fracture surface, and the maximum pressure of the posterior malleolar surface was 8.022 MPa. This study shows that reconstruction of the lateral malleolus in supination-external rotation ankle injuries significantly affects the stress distribution at the posterior malleolar joint surface. Through this reconstruction, the pressure distribution of the posterior malleolar joint surface can be significantly reduced.

## Introduction

Ankle fractures account for approximately 3.9% of all systemic fractures and are a common type of intra-articular fracture. Approximately 14% to 44% of these fractures impact the posterior malleolus, and they often result in ankle instability^1^. The posterior malleolus refers to the structures behind the fibular notch of the distal tibia, posterior tubercle (Volkmann tubercle), ankle sulcus and posterior colliculus of the medial malleolus. It is an integral part of the distal tibiofibular complex, increasing the contact area of the tibiotalar joint, reducing the pressure per unit area of the tibiotalar joint, preventing backwards movement of the talus, and supporting and maintaining the stability of the ankle joint^2,3^. Isolated posterior malleolar fractures rarely occur alone. Most posterior malleolar fractures are associated with lateral ankle fractures and ligament injuries around the ankle, especially supination-external rotation (SER) ankle fractures caused by severe rotation^4^. Without timely and appropriate treatment, the area is susceptible to traumatic arthritis, affecting the function of the ankle joint^5,6^.

In the treatment of supination-external rotation ankle joint injuries, anatomical reconstruction and rigid fixation of lateral ankle fractures are usually not difficult to carry out, but controversy exists regarding the type and effectiveness of fixation for posterior malleolar fractures^7^. Current opinion holds that some posterior malleolar fragments, although small, play an important role in maintaining the stability of the lower tibiofibular ligament^8^. Thus, exploring how the mechanical changes before and after external ankle reconstruction for the posterior malleolar facet directly affect the stability of the posterior malleolar fracture fragment and indirectly affect the lower tibiofibular ligament and overall stability of the ankle is essential. The final results of such an analysis may suggest new ideas for the treatment of posterior malleolar fractures in supination-external rotation-type injuries.

In this model, a three-dimensional model was constructed through the foot supination posture, and the setting of this posture was consistent with the foot posture of supination and external rotation injury. The injury patterns of SER was divided into four stages: stage I: tearing of the anterior ligament of the tibiofibular syndesmosis or avulsion fracture of the ligament attachment point; stage II: on the basis of the stage I injury, a fibula spiral fracture is added, and the fracture line is inclined from the posterior upper part to the anterior lower part; stage III: on the basis of the stage II injury, the posterior ligament of the tibiofibular joint is torn, or the ligament is avulsed at the attachment point of the posterior fibular tubercle, and there is an avulsion fracture at the attachment point of the tibia; stage IV: avulsion fracture of the medial malleolus or deltoid ligament tearing is added on the basis of the stage III injury. We used rupture of the anterior tibiofibular ligament to simulate a stage I injury of supination and external rotation; we simulated a stage II injury of supination and external rotation by constructing a fibular fracture line from the posterior upper part to the anterior lower part; and we simulated a stage III injury of supination and external rotation through rupture of the posterior tibiofibular ligament. In addition, a complete injury model of the lateral malleolus was developed for the stage III injury. After applying pressure, we explored and analysed the stress changes of the posterior malleolar surface.

Based on this study, we can determine how the integrity of the lateral malleolus affects the stability of the posterior malleolus and what kind of instability, if any, emerges. This is expected to help clinicians understand the mechanical changes after lateral malleolar reconstruction, remind them to pay attention to the rotational instability of the posterior malleolus and promote the solution of existing clinical problems. The study provides a biomechanical basis for fixation of the posterior malleolus after reconstruction of the lateral malleolus.

## Materials and methods

### Acquisition of ankle CT images

One healthy adult volunteer with no previous history of injury, such as ankle fracture or dislocation, or pathological conditions, such as ankle arthritis, bone disease or bone tumour, was selected. The right ankle joint of the volunteer (male, 60 kg and 28 years old) was imaged with thin-section CT, with a scanning layer thickness of 0.625 mm, resulting in 657 images of 512 × 512 pixels that were saved in DICOM format. During the CT scan, the volunteer's ankle was non-weight-bearing in the supination position. This study was conducted in keeping with the Declaration of Helsinki and has been reviewed and approved by the ethics committee of Jiading District Central Hospital Affiliated Shanghai University of Medicine &Health Sciences, all methods were carried out in accordance with relevant guidelines and regulations. Written informed consent was obtained from the healthy adult prior to enrollment in the study as per our study protocol reviewed and approval by local institutional review boards.

### 3D reconstruction and optimization

**Figure 1:**
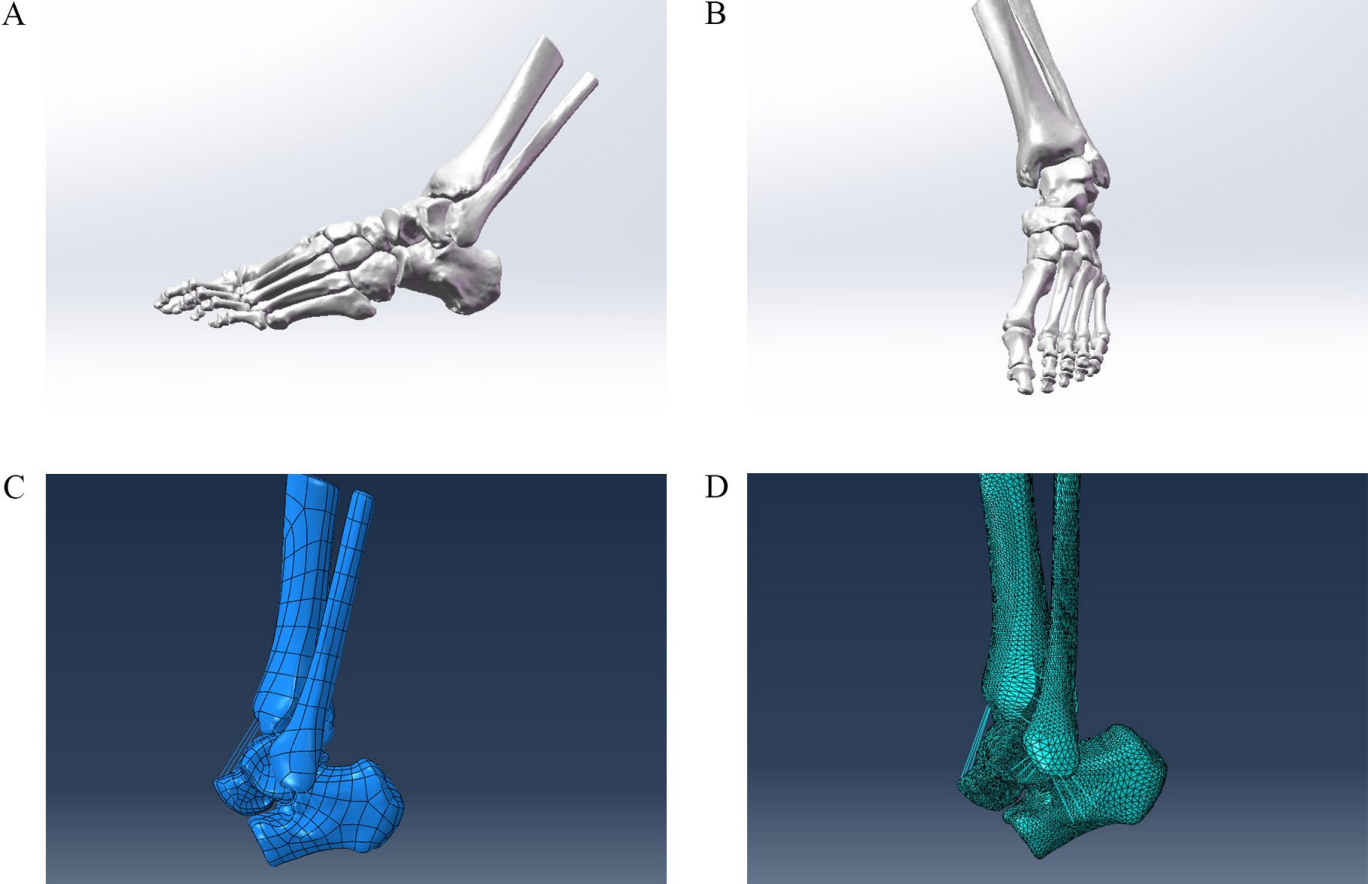
Process of establishing a finite element analysis model of a supination-external rotation ankle injury. (**A**, **B**) Surface Fitting. (**C**) the Finite element calculation grid model. (**D**) Calculation boundary.

DICOM format images were imported into Mimics 21.0 software (Materialise, Belgium); the images were segmented; and the tibia, talus, and fibula were reconstructed. Multiple bones (including the calcaneus, navicular cuneus, mediolateral cuneus, dice cuneus, and mediolateral cuneus) beneath the talus were fused to reconstruct their 3D models (Fig. 1). The following processes were carried out sequentially: (a) Automatic threshold segmentation and differentiation were carried out according to the grey value of different tissues. And the bone tissue was preliminarily separated. (b) A mask was used to establish the structure model of each part. (c) The manual layer editing tool was used to eliminate the redundant part or fill in the missing part. (d) The model was wrapped and smoothed. The hole and smooth surface were filled. The corresponding three-dimensional model was preliminarily established. And the model data file was exported in STL format.

We imported the STL format file generated by Mimics into Geomagic Studio 2017 software, erased the nails and redundant features of the model, and then smoothed the model. Then, the accurate surface module was used to detect the contour line of the model, any deformed or unreasonable contour line was edited, and an additional contour line was added appropriately to facilitate the generation of surface patches. After the surface patches were generated successfully, the surface was fitted, and the fitted model was exported to a general STEP format model data file.

### Establishment of the finite element model

We imported the optimized 3D models into SolidWorks software, carried out feature recognition and surface diagnosis on the geometric model, repaired any problematic surfaces, constructed an articular cartilage model by using the stretching and segmentation commands on the part interface, drew 3D lines in the 3D sketch to simulate the ligament model, and finally established a complete three-dimensional ankle model including the tibia, fibula, talus, calcaneus, cartilage and ligament and then saved the model as a 3D geometry file in X_T format. The ligaments we have established included the anterior tibiofibular ligament, posterior tibiofibular ligament, anterior talofibular ligament, posterior talofibular ligament, anterior tibiofibular ligament, posterior tibiofibular ligament, tibiocalcaneal ligament, calcaneal ligament and interosseous membrane. The obtained geometric models were imported into ABAQUS software (2018, Dassault, Providence, RI, USA) to build finite element models with the help of the attachment points and anatomical locations of ligaments determined from reference documentation. We used the tetrahedral mesh element C3D4. There were a total of 362,351 nodes and 261,420 units (Fig. 1). Bone and ligaments were idealized as isotropic^9^, homogeneous linear elastic materials, and the material parameters are listed in Table 1^10,11^. In brief, bonding contact was used between the ligament and bone, calcaneus and talus, while face-to-face contact was used for the talus, tibiofibula and calcaneus cartilage and bone. The friction formula was the penalty function algorithm, the normal contact stiffness mode was "hard" contact, and the friction coefficient was set to 0.2 to establish the finite element analysis model^12^.

**Table 1:** Material parameters^10,11^.

	Elastic modulus (MPa)	Poisson’s ratio
Bone	14,000	0.3
Cartilage	15	0.46
Ligament	260	0.49

### Research on grid convergence

The normal supination and external rotation ankle joint model was divided into grids of different levels. The grid levels were divided into coarse, semi-coarse, fine and very fine. Then, the same boundary conditions and loads were applied to the four grid models. The four grid models all used the same boundary conditions and loads for analysis. The boundary conditions were a fixed calcaneus and talus, and the load was a self-weight load of 600 N. The stress and displacement results obtained from the analysis were shown in the table below (Table 2).

**Table 2:** Research on grid convergence.

Grid level	Element quantity	Max. stress (MPa)
Coarse	143,507	68.5
Semi coarse	201,317	95.55
Fine	264,023	83.73
Very fine	369,803	77.76

The stress results of the fine grid level were similar to those of the very fine grid level. The stress distribution calculated by the fine mesh level and the very fine mesh level was the same. The stress values were similar, and the maximum stress position was on the tibia. So the subsequent analysis adopted the fine grid model as the finite element analysis model based on a comprehensive consideration of calculation accuracy and calculation time.

### Verification and analysis of the finite element model

In this part, the model loading parameters were based on the fixed lower surface of the talus. A dead weight loaded between the tibia and proximal fibula and an internal rotation moment were used to simulate a post rotation-external rotation-type injury. Three directional fixation restraints were set with full degrees of freedom in XYZ at the under-surface of the talus, and a reference point was established near the upper surface of the tibia and fibula, coupled with the upper surface degrees of freedom.

**Figure 2:**
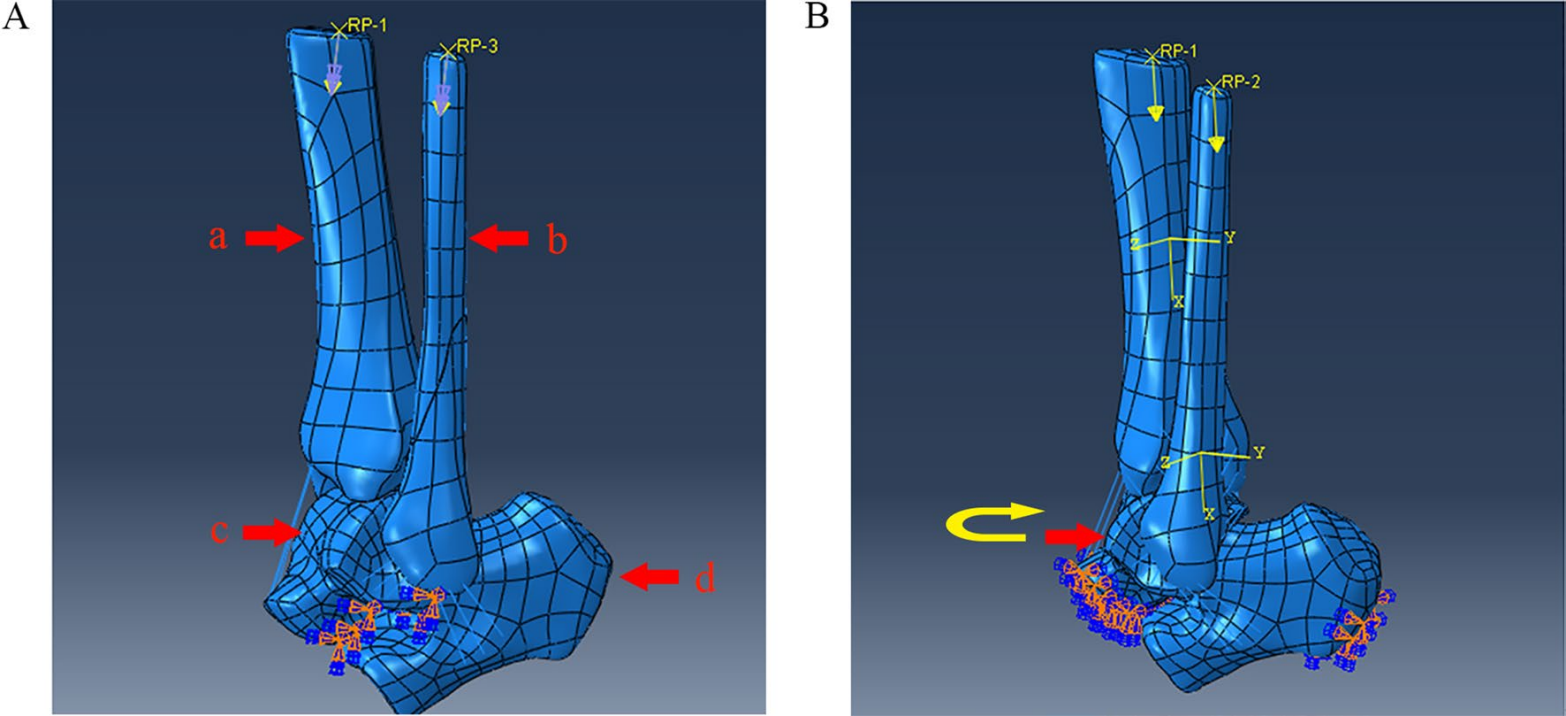
Boundary constraints and load diagram of the damage model analysis. (**A**) Boundary constraints. (**B**) The load conditions set. a: Tibia. b: Fibula. c: Talus. d: Calcaneus. The yellow arrow indicates the internal rotation moment to the talus.

The FEM simulated the load distribution of the tibia and fibula when a human was standing on one foot^13^, with application of a dead-weight load (480 N compression on the upper surface of the tibia and 120 N compression on the upper surface of the fibula) and an internal rotation moment (gradually increasing internal rotation moment, simulating a stage III injury). A 600 N vertical compressive load was applied to the upper sections of the lower tibia and fibula of the model, where the calcaneus was fixed and the talus was constrained (Fig. 2). In this way, the validity of the model was analysed.

**Figure 3:**
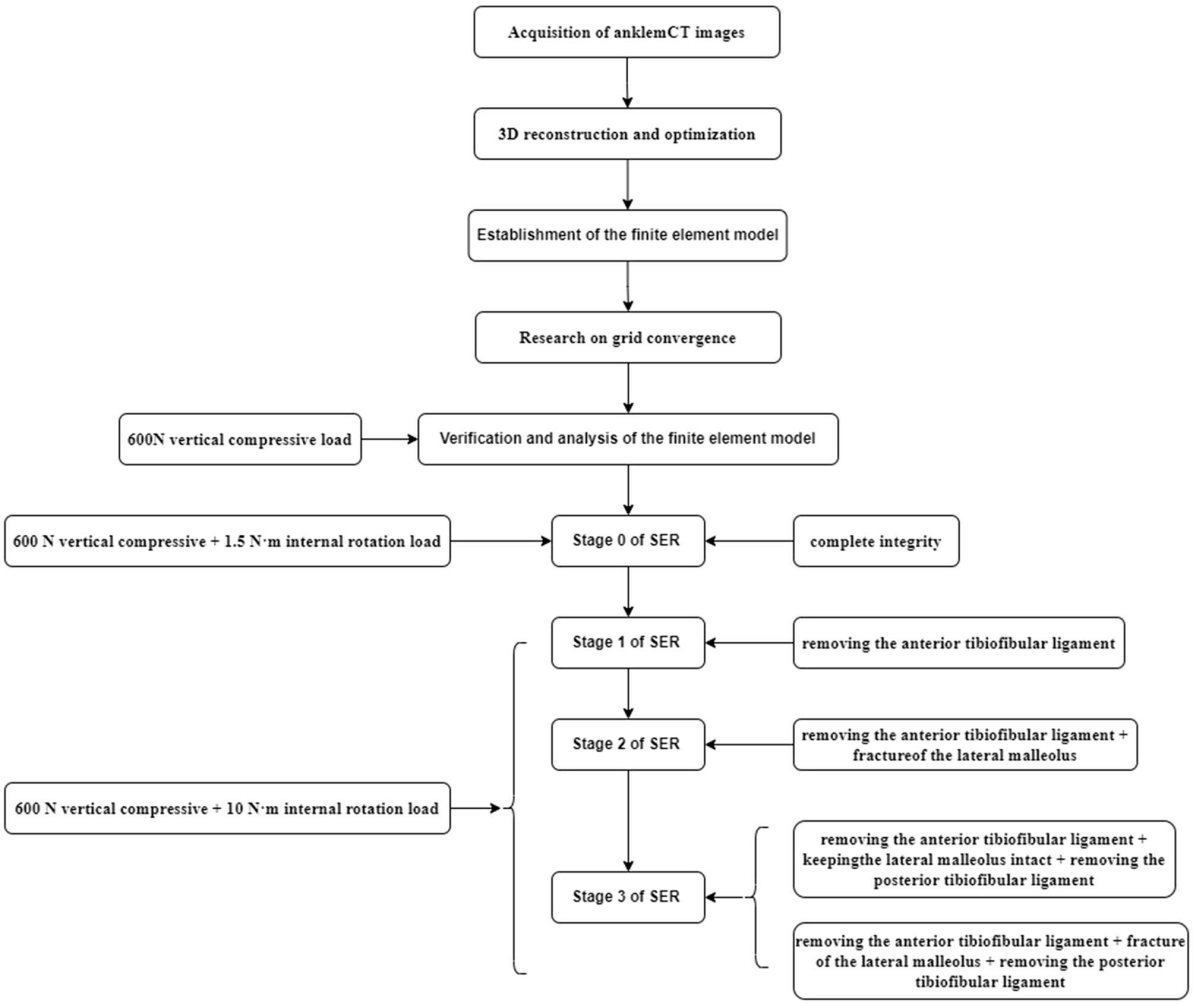
Flow chart of this study.

And all the above studies followed Fig. 3.

### Ethics approval and consent to participate

This study was conducted in keeping with the Declaration of Helsinki and has been reviewed and approved by the ethics committee of Jiading District Central Hospital Affiliated Shanghai University of Medicine & Health Sciences, all methods were carried out in accordance with relevant guidelines and regulations. Written informed consent was obtained from the healthy adult prior to enrollment in the study as per our study protocol reviewed and approval by local institutional review boards.

## Results

### Verification of the finite element model

**Figure 4:**
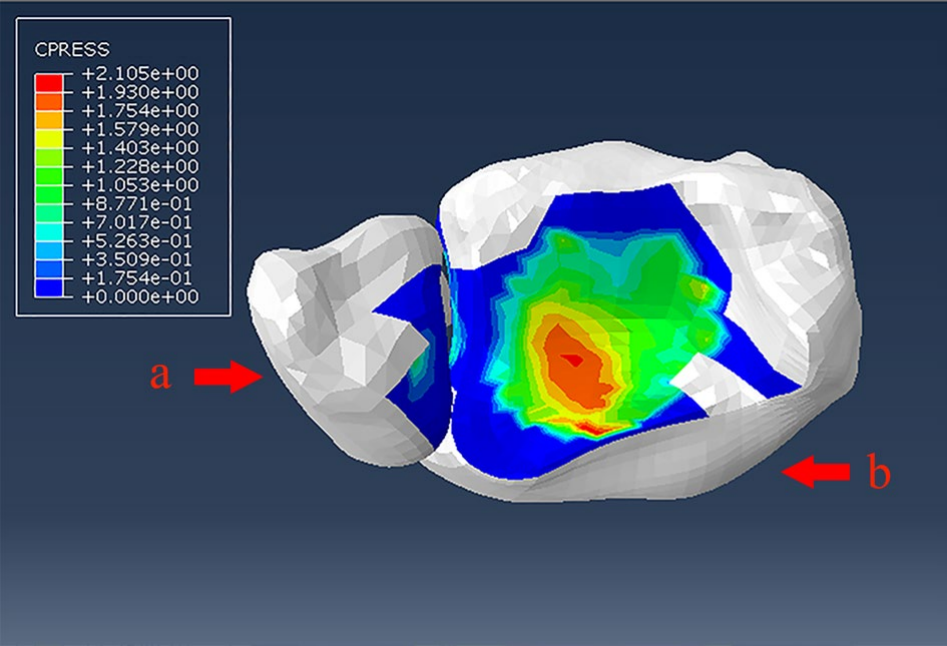
Validation of model validity by finite element analysis of a supination-external rotation ankle injury. a: Tibia. b: Fibula.

All the data were shown in Table 3. The maximum contact stress of the ankle joint surface was 2.105 MPa. The location of maximum contact stress (2.105 MPa) was the forward middle of the tibiotalar joint surface. The contact area was 373.658 mm^2^ (Fig. 4). The maximum pressure of the contact surface of this model was close to the results of Anderson's experiment on the ankle. And the pressure had a similar distribution, indicating that the model constructed in this study was consistent with Anderson's research results. And the model was validated as effective^9^. The calculations of outcomes were based on the maximum stress location and pressure on the articular surface.

**Table 3:** Results of the finite element analysis.

Finite element model	Damage mode	Load	Location of maximum stress	Maximum stress	Maximum contact stress	Location of maximum contact stress	Figure
Verification and analysis	–	600 N vertical compressive load	–	–	2.1059 MPa	Posterior malleolar surface	Figure 4
Stage 0 of SER	Complete integrity	600 N vertical compressive + 1.5 N m internal rotation load	Anterior tibiofibular ligament to the fibula	51.05 MPa	2.549 MPa	Posterior malleolar surface	Figure 5A,B
Stage I of SER	Removing the anterior tibiofibular ligament	600 N vertical compressive + 10 N m internal rotation load	Posterior tibiofibular ligament to the tibial	271.2 MPa	2.626 MPa	Posterior malleolar surface	Figure 6A,B
Stage II of SER	Removing the anterior tibiofibular ligament + fracture of the lateral malleolus	600 N vertical compressive + 10 N m internal rotation load	Fibula fracture surface	82 MPa	7.787 MPa	Posterior malleolar surface	Figure 8A,B
Stage III of SER	Removing the anterior tibiofibular ligament + keeping the lateral malleolus intact + removing the posterior tibiofibular ligament	600 N vertical compressive + 10 N m internal rotation load	Posterior talofibular ligament	132.7 MPa	4.505 MPa	Fibula	Figure 9A,B
	Removing the anterior tibiofibular ligament + fracture of the lateral malleolus + removing the posterior tibiofibular ligament	600 N vertical compressive + 10 N m internal rotation load	Fibula fracture surface	82.72 MPa	8.022 MPa	Posterior malleolar surface	Figure 10A,B

### Model of a stage I supination-external rotation ankle injury

**Figure 5:**
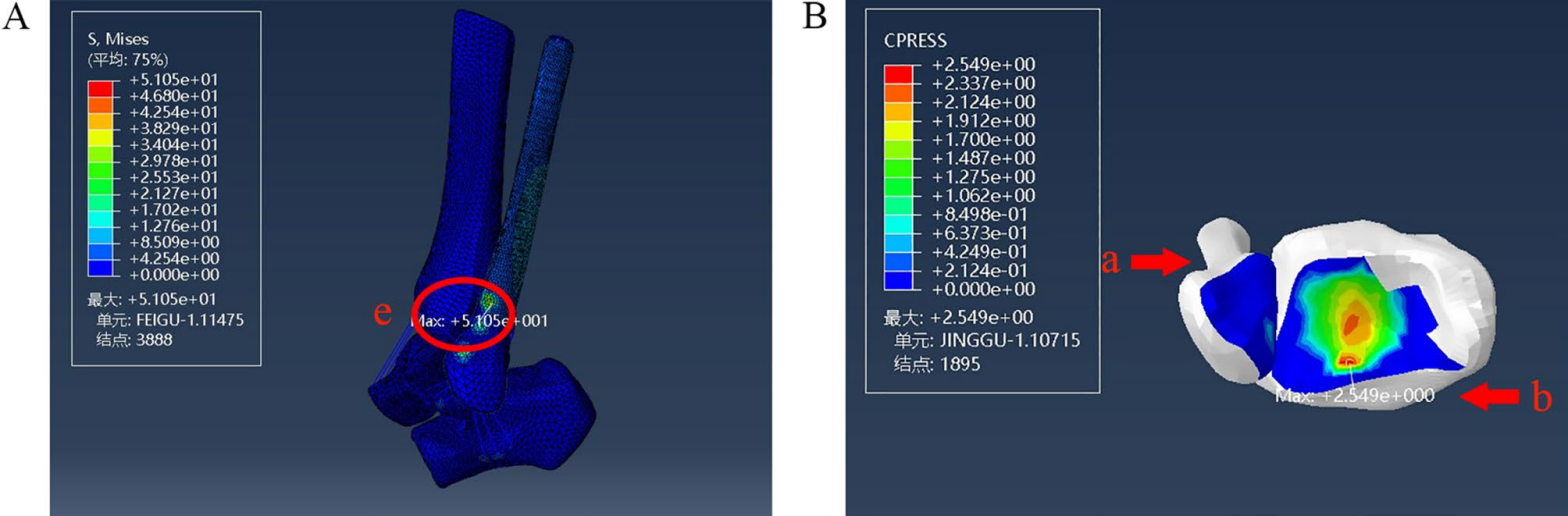
Stress distribution during a stage I supination-external rotation ankle injury. (**A**) Maximum stress view. (**B**) Maximum contact stress view. Units of measurement: Mpa. a: Tibia. b: Fibula. e: The anterior tibiofibular ligament.

The maximum value of stress at 1.5 N m internal rotation was 51.05 MPa at the attachment point of the anterior tibiofibular ligament to the fibula, and the maximum value of pressure at the posterior malleolar surface was 2.549 MPa (Fig. 5). This was consistent with the description of Lauge-Hansen typing in the clinic^14–16^.

Lauge–Hansen typing in the clinic could be divided into four stages: stage I: tearing of the anterior ligament of the tibiofibular syndesmosis or avulsion fracture of the ligament attachment point. The anterior tibiofibular ligament was the earliest injury in the Lauge–Hansen injury. At this time, the ligament was under the greatest stress, which was consistent with the clinical and experimental results.

**Figure 6:**
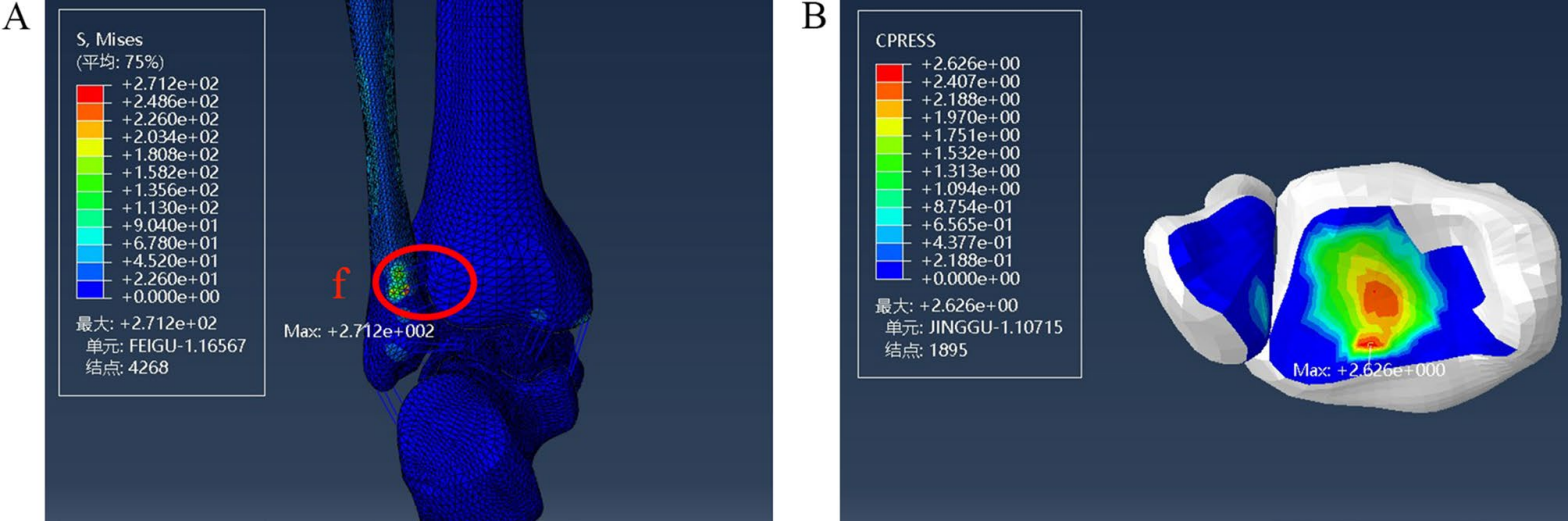
Distribution of stress during injury to the anterior tibiofibular ligament in a supination-external rotation ankle injury. (**A**) Maximum stress view. (**B**) Maximum contact stress view. Units of measurement: Mpa. f: The posterior tibiofibular ligament.

Upon removing the anterior tibiofibular ligament, the lateral malleolus remained intact. At this time, we continued to load a 10 N m internal rotation moment. The corresponding maximum stress (271.2 MPa) was located at the tibial attachment point of the posterior tibiofibular ligament, and the maximum pressure of the posterior malleolar surface was 2.626 MPa (Fig. 6).

### Model of a stage II supination-external rotation ankle injury

**Figure 7:**
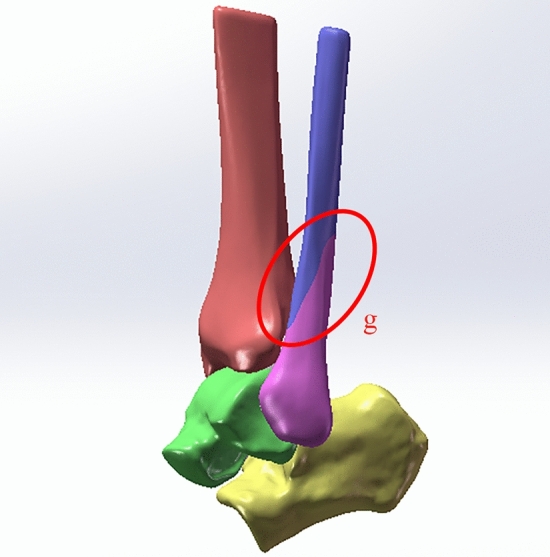
Model of lateral malleolar fracture in a supination-external rotation ankle joint injury. g: The fracture line.

**Figure 8:**
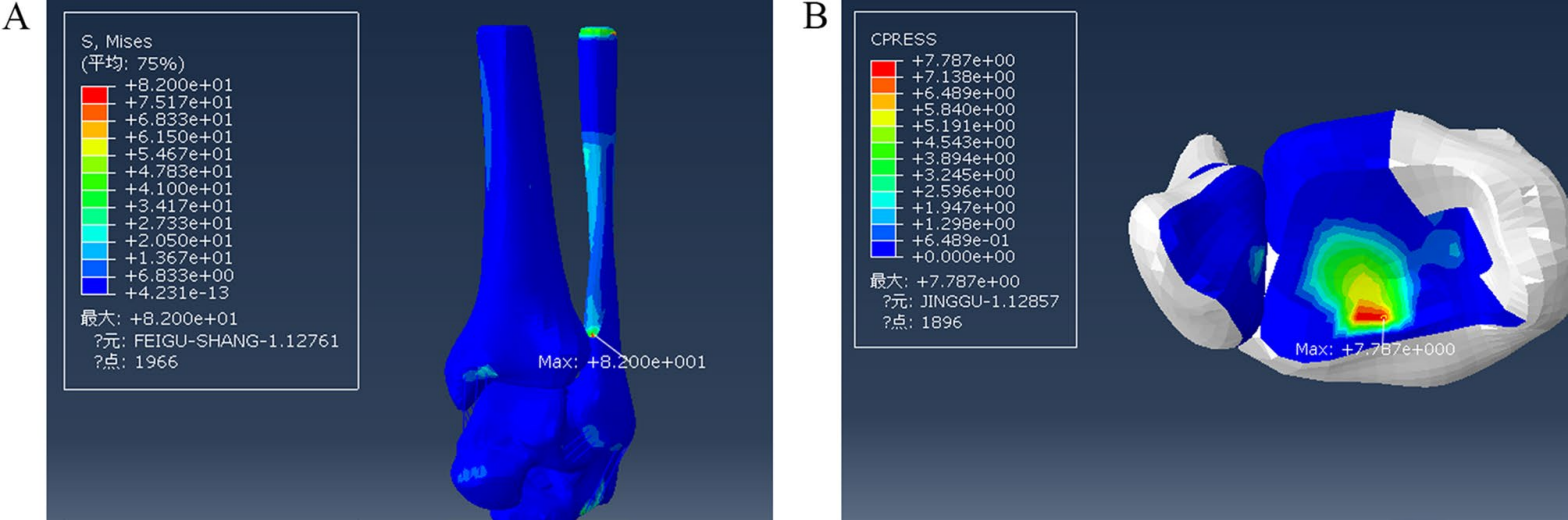
Distribution of stress during injury of the lateral malleolar fracture in a stage II supination-external rotation ankle injury. (**A**) Maximum stress view. (**B**) Maximum contact stress view. Units of measurement: Mpa.

The fibula fracture line was drawn posterosuperior and inferior to construct the stage II injury fracture model (Fig. 7). Fracture lines needed to be cut out in 3D software to establish a fibular fracture model. The von Mises stress and contact pressure were evaluated. The calculation showed that the maximum stress on the fibula fracture surface when a 10 N m internal rotation moment was applied was 82 MPa; the maximum pressure at the posterior malleolar surface was 7.787 MPa (Fig. 8).

Damage to the supination and external rotation developed from stage I to stage II. Under the same load, the maximum stress changed from the tibial attachment point of the posterior tibiofibular ligament to the fracture end of the lateral malleolus. The maximum pressure of the posterior malleolar surface increased from 2.626 to 7.787 MPa. The location of the highest stress of the posterior malleolus moved outward significantly.

### Model of a stage III supination-external rotation ankle injury

**Figure 9:**
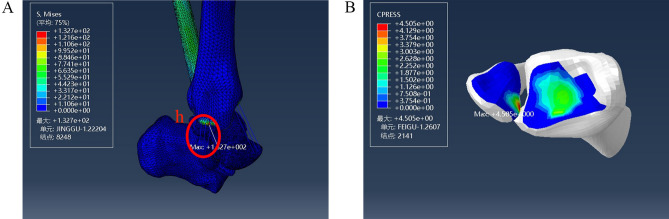
Distribution of stress during injury of the posterior tibiofibular ligament in a stage III supination-external rotation ankle injury. (**A**) Maximum stress view. (**B**) Maximum contact stress view. Units of measurement: Mpa. h: The posterior talofibular ligament.

With the lateral malleolus intact, a model of a stage III injury was established by removing the posterior tibiofibular ligament. Loading with internal rotation was continued; the maximum stress after removing the posterior tibiofibular ligament was 132.7 MPa, located at the attachment point of the posterior talofibular ligament to the fibula, and the maximum pressure on the fibula was 4.505 MPa (Fig. 9).

**Figure 10:**
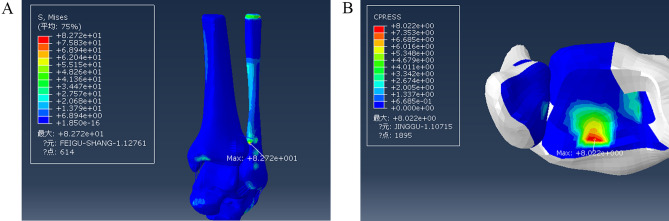
Stress distribution during external ankle fracture associated with injury to the posterior tibiofibular ligament in a stage III supination-external rotation ankle injury. (**A**) Maximum stress view. (**B**) Maximum contact stress view. Units of measurement: Mpa.

On this basis, fibula fracture lines were drawn from the posterior upper part to the anterior lower part to construct a fracture model (Fig. 7). The maximum stress was 82.72 MPa, located at the fibula fracture surface, and the maximum value of pressure at the posterior malleolar surface was 8.022 MPa (Fig. 10).

The damage of supination and external rotation developed from stage II to stage III. Under the same load, the positions of maximum stress, which were all fibular fracture surfaces, did not change. The maximum pressure of the posterior malleolar surface increased from 7.787 to 8.022 MPa. The maximum stress position of the posterior malleolus did not change significantly.

In the case of a stage III injury with an intact lateral malleolus, under the same load, the maximum stress was located at the fibular attachment point of the posterior peroneal ligament. The maximum pressure of the posterior malleolar surface decreased significantly to less than 4.505 MPa. There was no prominent stress concentration in the stressed part of the posterior malleolus.

## Discussion

Lauge–Hansen’s classification, which was published in a 1950 issue of Archives of Surgery, has become one of the most widely used ankle fracture classification systems. In that article, Niel Lauge–Hansen presented an ankle fracture classification and an explanation for low-energy fractures caused by severe rotation of the ankle^15^. This classification is based on the foot position at the time of the traumatic event (supination or pronation) and the direction of the deforming forces (abduction, adduction, or external rotation). Seventy percent of these fractures were of the supination-external rotation type: the foot was in the supination position due to external severe rotation. Stage III–IV damage involves bony structures or associated ligaments of the posterior malleolus^17^.

Through three-dimensional heat map analysis, Yu Tao et al. found that most of the fracture lines of the posterior malleolus were concentrated in an arc-banded region that started from 1/7 to 2/7 of the tangent line of the posterior edge and ended at 5/11 to 7/11 of the tangent line of the outer edge^18^. The proportion of posterior malleolar fracture fragments to the total articular surface of the distal tibia was 14.96%^19^. In the past, a posterior malleolar fracture area of more than 25% was considered to be an indication for surgical treatment of posterior malleolar fracture. Verhage et al. found that the incidence of osteoarthritis was approximately 48% when the area of posterior malleolar fracture accounted for 5–25% of the tibial articular surface and as high as 54% when the area was greater than 25%, and suitable reduction could not be maintained except by internal fixation^5,20^.

Mangnus et al. concluded that the position of the posterior malleolar fracture line may have a greater impact on stability than the size of the fracture area; even slight posterior malleolar fracture affects ankle joint stability^21^. Gardner et al. confirmed on postoperative CT that posterior malleolar fracture affects the stability of the inferior tibiofibular ligament and that unfixed posterior malleolar fracture can lead to tibiofibular joint subluxation^22,23^. In recent years, some studies have found that fixation of posterior malleolar fracture fragments can restore the tension of the lower tibiofibular posterior ligament and improve the reduction in the lower tibiofibular syndesmosis^24^. The stability obtained is better than that of lower tibiofibular screw fixation. Therefore, some scholars have even reported that in the case of posterior malleolar fracture, no matter how large the fracture fragment is, anatomical reconstruction should be carried out to reduce the use of lower tibiofibular screws^25^. Therefore, for the treatment of posterior malleolar fractures, determining whether surgery is necessary by the size of the bone only is not reliable.

Based on the data in our experiment, it can be concluded that anatomical reconstruction and rigid fixation of the lateral malleolus can significantly reduce the stress of the posterior malleolus in supination and external rotation ankle injury. Furthermore, the completion of anatomical reconstruction moves the maximum distribution of stress change from the broken end of the fracture to the ligament attachment point, and the prominent concentration of the maximum stress part of the posterior malleolar clearly disappears. The disappearance of prominent stress concentrations in the posterior malleolus is consistent with the phenomenon of self-reduction of posterior malleolar fractures after lateral malleolar reduction and reconstruction. Therefore, for posterior malleolar fractures, the establishment of rotational stability may be more important than vertical stability. If the stability of the posterior malleolar joint and posterior tibiofibular ligament is not satisfactory, the maximum contact stress of the posterior malleolar surface will increase to more than twice that of the uninjured model, which will further aggravate the instability of the ankle joint. Therefore, in supination-external rotation ankle injury, the integrity of the lateral malleolus has an important impact on the stability of the posterior structure of the ankle, which coincides with the views of Mangnus, Gardner and others^21,22^. Regarding the choice of internal fixation method for posterior malleolar fracture, two screws and gaskets provide good anti-rotation capacity, while buttress plates have better anti-vertical-motion capacity. Therefore, the selection of two screws and gaskets for internal fixation may be sufficient in the case of posterior malleolar fracture in supination and external rotation ankle injury after lateral ankle reconstruction, but this needs to be proven by further biomechanical research.

A limitation of this study is that the simplified simulation model differs from the actual situation. The ligament is not a linear elastic material, and its displacement load curve presents multiphase characteristics. In different pretension states, its stiffness is different. In the state of supination lateral preload, the material parameters of the external collateral ligament may be different from those of other ligaments in the simplified simulation model of finite element simulation. Moreover, no research has been conducted on Stage IV injuries involving triangular ligament and medial malleolar fractures, the construction of posterior malleolar fractures or the selection of internal fixation.

## Conclusion

This study shows that the external ankle integrity of supination-external rotation ankle injury is of great significance to stress changes at the posterior malleolar surface. When the lateral malleolus is intact, the pressure distribution on the posterior malleolar surface can be significantly reduced, and the disappearance of stress concentrations is helpful to reduce the stress associated with posterior malleolar fracture. At this time, the rotational stress imposed by posterior malleolar fracture is significantly higher than the vertical stress, and clinicians should pay more attention to the former in treatment.

## Data Availability

The datasets generated and/or analysed during the current study are not publicly available due to patient personal health confidentiality agreement but are available from the corresponding author on reasonable request.
