# Author Correction: Establishment of a finite element model of supination-external rotation ankle joint injury and its mechanical analysis

**DOI:** 10.1038/s41598-022-26539-7

**Published:** 2022-12-20

**Authors:** Xin Zhang, Pinliang Xie, Weirong Shao, Ming Xu, Xiaoping Xu, Yong Yin, Lan Fei

**Affiliations:** Department of Orthopedics, Jiading District Central Hospital Affiliated Shanghai University of Medicine & Health Sciences, 1 Chengbei Road, Shanghai, 201800 China

Correction to: *Scientific Reports* 10.1038/s41598-022-24705-5, published online 22 November 2022

The original version of this Article contained errors in Table 3, where the order of the figure citations in the column “Figure” was incorrect.

The correct and incorrect values appear below.

Incorrect:

**Table:** 

Figure
Figure 3
Figure 4A,B
Figure 5A,B
Figure 7A,B
Figure 8A,B
Figure 9A,B

Correct:

**Table:** 

Figure
Figure 4
Figure 5A,B
Figure 6A,B
Figure 8A,B
Figure 9A,B
Figure 10A,B

The original Article has been corrected.

